# Commencement Exercises

**Published:** 1902-06

**Authors:** 


### COMMENCEMENT EXERCISES.

### ONTARIO VETERINARY COLLEGE.

THE closing exercises of the session of 1901–1902 of this college were held in the college buildings, Temperance Street, March 27, 1902. The Principal, Professor A. Smith, F.R C.V.S., took the chair, and with him on the platform were Mayor Howland, Profs. Mavor and Laing, of the University of Toronto; Mr. W. K. McNaught, President of the Toronto Industrial Exhibition Association, and Dr. Duncan. Professor Smith opened the meeting by a short address, and called on Dr. Duncan to read the graduating and honor lists, also the list of prize winners.

Mayor Howland, in a short address that was received with applause by the students, gave much credit to Professor Smith for organizing the college and carrying it on so successfully for so many years. He also spoke in laudatory terms of the veterinary profession and its benefits to the country at large. Professor Mavor, Professor Laing, and Mr. W. K. McNaught also gave short addresses that were greeted with cheers by the students.

In closing the meeting Mr. Smith warmly thanked the students for the splendid picture of the graduating class and staff of the college presented to him by the students. He also remarked that two prominent members of this year's graduating class had recently seen service in South Africa, and that three more of this year's graduates would probably shortly proceed to the seat of war, there to assist in upholding the honor of the British flag.

The following is the list of graduates and prize winners:

F. Rudolph Adams, Cardiff, Wales; Fred. W. Anderson, Buffalo, N. Y.; Henry M. Armour, Warsaw, N. Y.

Harry K. Berry, Paterson, N. J.; Thomas A. Blacklock, Campbellville; Christopher J. Bousfield, Toronto; Phineas Bridge, Paterson, N. J.; Walter T. Brophy, Montevideo, Minn.; Jared Burton, Wheaton, Minn.

Gilbert F. Candage, Bluehill, Maine; Joseph E. Carter, Riverhead, Long Island, N. Y.; Nels A. Christianson, Magnolia, Minn.; Matthew G. Connolly, Sundridge; Clarence J. Cooper, Warwick, Bermuda, W. I.; Thomas F. Colling, Toronto.

James Morgan Dand, Deloraine, Man.; John B. Darling, South Peacham, Vermont; Charles E. Dille, Ville Ridge, Ill.; Charles H. Doyle, Summerside, P. E. I.; Lawrence L. Doyle, Summerside, P. E. I.

Bert C. Eldredge, Tedrow, Ohio.

D. Alex. Fasken, Paris; Edward Roy Farewell, Drayton; W. Francis Forest, Hicksville, Ohio; Ralph Edward Freeman, Rockland, Maine.

William A. Gill, Verschoyle; G. Arthur Gohn, Toronto.

Charles L. Hayward, Georgetown, Ill.; Demerest T. Havens, Manasquan, N. J.; George A. Harvey, Cleveland, Ohio; Joseph S. Hollingsworth, La Salle, Ill.; Robert A. Hume, Watford; Gardiner Harvey, Guelph.

Wesley I. Irwin, Little Britain.

James W. Jackson, Ventnor; T. Fred. Johnston, St. John, N. B.

T. F. Kimball, Elmore, Ohio.

John T. Leslie, Flora, Ind.

W. D. MacCormack, Enterprise; James A. McLeish, Arkona.

Edward J. Magee, Warrenburg, N. Y.; Milton M. Marshall, Cochranton, Pa.; Walter L. Mills, Warsaw, N. Y.; John P. Molloy, Rosser, Man.; H. Clifford Murray, South Glen Falls, N. Y.

William J. Neil, Omemee; R. C. M. Nyblett, Strathclair, Man.

Francis Vincent Perry, Regina, N. W. T.; Clarence Clement Petty, Hastings, Mich.; John Harland Pickering, Forest; Charles Edgar Poe, Leitersburg, Md.; Frank W. Powell, Akron, Ohio.

Charles R. Query, Jackson, Mo.

Shearman Ransom, Westholm, B. C.; Herbert E. Rea, St. Mary's; W. E. E. Robbins, Halifax, N. S.

George L. Schneider, Canton, Ohio; Omar O. Selle, Cameron, Mo.; J. Clarence, Singer, Perth Amboy, N. J.; Llewellyn Snyder, Huntsville; Chauncey C. Stevens, Yale, Mich.; A. Newton Stewart, Waterloo, Iowa; Clark A. Stewart, Waterloo, Iowa; Robert Stewart, St. John's, Newfoundland; Theodore J. Stover, Norwich; John Henry Sturm, Chilton, Wis.; Lorne Daniel Swenerton, Carberry, Man.; William F. Schwiesow, Columbus, Wis.

Arthur R. Torrie, Chatsworth.

R. Thomas Williams, Blackinton, Mass.

W. C. Van Allstyne, Red Creek, N. Y.

Disease and Treatment. Seniors: First prize, S. Ransom, silver medal; second prize, R. C. M. Nyblett; third prize, C. R. Query and J. Pickerton, equal.

Honors: Brophy, Burton, Cooper, Colling, Dand, Freeman, Johnston, G. A. Harvey, Hume, Hollingsworth, Irwin, Kimball, McLeish, Molloy, Murray, Neil, Poe, Powell, Schwiesow, Schneider, O. O. Selle, C. Stevens, Stover, Sturm, Van Allstyne.

Materia Medica. Seniors: First prize, J. H. Pickering; second prize, E. E. Robbins; third prize, J. A. McLeish.

Honors: Adams, Bowsfield, Blacklock, Colling, Candage, Connolly, Dand, Farewell, Freeman, Forest, Hume, G. A. Harvey, Hollingsworth, Murray, Neil, Nyblett, Ransom, C. A. Stewart, Singer, Stover, Sturm, Van Allstyne.

Chemistry. Seniors: First prize, J. H. Pickering; second prize, S. Ransom; third prize, R. M. Nyblett.

Honors: Adams, Armour, Burton, Dand, Farewell, G. A. Harvey, Jackson, Murray, Singer, Williams.

Pathology. Seniors: First prize, S. Ransom; second prize, J. H. Pickering; third prize, R. M. Nyblett.

Honors: C. J. Bousfield, T. A. Blacklock, P. Bridge, T. Colling, P. Crerar, J. B. Darling, J. M. Dand, E. Farewell, D. T. Havens, J. S. Hollingsworth, R. A. Hume, G. A. Harvey, J. A. McLeish, F. P. Molloy, E. E. Robbins, L. Swenerton, C. A. Stewart, T. Stover, O. O. Selle, C. Singer, C. C. Stevens, A. Torrie, W. C. Van Allstyne, J. E. Wurm.

Physiology. Seniors: First prize, T. J. Stover; second prize, R. C. M. Nyblett; third prize, E. E. Robbins.

Honors: Armour, Adams, Brophy, Connolly, Dand, G. A. Harvey, McLeish, Molloy, Pickering, Ransom, Wurm, Williams.

Anatomy. Seniors: E. E. Robbins, first prize, silver medal; R. M. Nyblett, second prize; W. C. Van Allstyne, third prize.

Honors: H. W. Armour, Jared Burton, T. A. Blacklock, H. Berry, C. J. Bousfield, G. F. Candage, P. Crerar, J. M. Dand, J. B. Darling, R. E. Freeman, G. A. Harvey, J. S. Hollingsworth, R. A. Hume, J. W. Jackson, J. P. Molloy, H. C. Murray, J. A. McLeish, W. J. Neil, F. W. Powell, J. H. Pickering, C. R. Query, S. Ransom, O. O. Selle, C. Stevens, T. J. Stover, W. T. Schwiesow, A. D. Swenerton, G. L. Schneider, J. E. Wurm, R. T. Williams.

Entozoa. Seniors: G. A. Harvey, prize.

Honors: T. A. Blacklock, Jared Burton, N. A. Christianson, J. M. Dand, C. H. Doyle, E. R. Farewell, J. W. Jackson, Wesley Irwin, T. F. Kimball, E. J. Magee, H. C. Murray, James I. McLeish, R. M. Nyblett, J. H. Pickering, E. E. Robbins, S. Ransom, W. T. Schwiesow, L. D. Swenerton, A. P. Torrie, W. C. Van Allstyne, J. E. Wurm.

Dissected Specimens. Gold medal given by the Toronto Industrial Association, awarded to Jared Burton; second prize, F. W. Powell.

Best General Examination. Gold medal given by the Ontario Veterinary Association, awarded to R. M. Nyblett.

Honors: J. M. Dand, R. E. Freeman, G. A. Harvey, J. H. Pickering, C. R. Query, E. E. Robbins, S. Ransom.

Primary Examinations. Anatomy: W. G. Chrisman, P. Crerar, F. J. Delaine, E. L. Fryer, H. W. Nobles, W. Thompson, J. E. Wurm.

Materia Medica: E. L. Fryer, M. S. Suttle, W. Thompson, J. E. Wurm.

Disease and Treatment. Juniors: First prize, C. B. Kern; second prize, F. Lambie; third prize, J. H. Part, E. H. Ives, C. A. Leslie, equal.

Honors: Bauer, Brenton, Busselle, Bowes, Chamberlain, Chorlton, Coates, Crawford, Drew, Fawcett, Fernandez, Fraser, Gillan, Gondey, Haffer, Harburn, Harris, Hartman, Henderson, Lee, Archie McMillan, McLevy, Mann, Meany, Mitchell, Monell, Oliver, Owens, Reinhardt, Richard, Rusk, Schweinler, Sprunger, Strive, Sykes, H. J. Taylor, J. C. Taylor, L. Thurston, Tomlinson, Van Vlaadern, Walker, Webb, Wilson, Williams.

Anatomy. Juniors: First prize, silver medal, C. A. Leslie; second prize, C. B. Kirn; third prize, T. B. Harris.

Honors: Willis L. Brenton, A. W. Busselle, G. A. Coates, J. T. Chorlton, A. H. Chamberlain, D. H. Chase, J. E. Crawford, D. B. Frazer, R. H. Gondey, J. W. Haffer, E. H. Ives, F. Lambie, L. G. Mitchell, W. G. McLevy, B. A. Owens, J. H. Part, Thos. Quinn, J. C. Rusk, E. Sutter, E. R. Struve, T. O. Sykes, H. A. Taylor, J. R. Tomlinson, E. A. Watson, Howard Williams, G. C. Webb.

Physiology. Juniors: First prize, silver medal, J. H. Part; second prize, C. B. Kern; third prize, F. B. Lambie.

Honors: Busselle, Crawford, Frazer, Hartman, Henderson, Ives, Leslie, H. J. Taylor, E. A. Watson.

Chemistry. Juniors: J. H. Part, first prize; F. B. Lambie, second prize; H. J. Taylor, third prize.

Honors: Busselle, Bulgin, Crawford, Coates, Chamberlain, Drew, Fernandez, Flowers, Fraser, Henderson, Ives, Leslie, Madill, Alexander McMillan, Vincente Ocampo, Sibley, Sykes, Walker, Watson.

Pathology. Juniors: T. B. Lambie, first prize; J. H. Part, second prize; C. A. Leslie, third prize.

Honors: G. Bulgin, W. L. Brenton, T. Bowes, A. W. Busselle, A. Chamberlain, J. Crawford, G. A. Coates, A. P. Drew, R. G. Flowers, A. Fernandez, D. Fraser, R. H. Gondey, H. Graham, H. Houze, W. Hartman, J. Haffer, W. Henderson, T. B. Harris, E. Ives, C. B. Kern, W. J. Lee, Alexander McMillan, R. S. Mack, Archie McMillan, Wm. Madill, T. J. Mitchell, J. K. Mann, B. Owens, L. Ocampo, E. Pallister, T. Quinn, W. H. Russell, T. Richards, A. A. Reinheardt, J. Rusk, W. A. Sparling, E. Struve, T. O. Sykes, H. W. Scheurer, F. Schweinler, R. Sprunger, G. R. Tomlinson, H. J. Taylor, S. West, H. Williams, G. C. Webb, W. M. Walker, E. Watson, C. Van Vlaanderen.

### CHICAGO VETERINARY COLLEGE.

The annual banquet given to the Faculty and students by the Trustees of the Chicago Veterinary College was held at the Sherman House, Chicago, on March 13, 1902. Prof. A. H. Baker presided. Prof. L. A. Merillat acted as toastmaster, and the programme was very elaborate. Mr. W. F. Hoehner, senior student, responded to the toast of "The Future of the Profession." Mr. E. L. Lewis, junior student, responded to the toast "Phases of College Life," and Mr. E. A. Rein, freshman, to the toast "Veterinary Education from the Stand-point of a Freshman." Musical numbers were furnished by the class quartette—Messrs. Hisgen, Parks, Perkins, and Axby—consisting of songs, piano selections, etc. Numerous toasts were responded to by the different members of the Faculty.

### Commencement Exercises.

The eighteenth annual commencement exercises of the Chicago Veterinary College were held at the Chicago Auditorum on Friday afternoon, March 28, 1902. Numerous friends of the graduating class and the Faculty were present. Prof. Joseph Hughes presided, and, addressing the class, stated that the session concluded was one of the most successful in the history of the college, the number of matriculates being 164. He said that in the class of this year, besides the students who have taken the complete course at this school, there were graduates and advanced students from six other colleges, and this, naturally giving rise to more or less friendly competition, proved a decided stimulus to the class as a whole in the pursuit of their studies. He congratulated them on their splendid attainments, whether judging them from their ordinary scholastic education or from the amount of technical veterinary knowledge which they possessed. Concluding, he expressed his admiration—an admiration shared by every member of the Faculty—of the deportment and general conduct of the class during their attendance. On behalf of the Trustees, he thanked the Faculty for the highly efficient manner in which they conducted their various classes, directly contributing to the success of the session. He then announced the names of the gentlemen who successfully passed the final examination, as follows:

B. F. Barber, Glidden, Iowa; C. Baynes, Jersey City, N. J.; J. W. Beckwith, Shullsburg, Wis.; R. J. W. Briggs, Garner, Iowa; W. W. Bronson, Wyoming, Iowa; J. W. Bunker, New Providence, Iowa; L. C. Butterfield, Marseilles, Ill.; F. W. Brewer, Indianapolis, Ind.; E. G. Cluts, Canton, Ill.; Charles J. Dawdy, Greenville, Ill.; H. Devitt, Chicago, Ill.; L. L. Diller, Marshalltown, Iowa; C. E. Dornheim, Pomfret Centre, Conn.; F.

Eckert, Reeseville, Wis.; J. E. Frank, Sandyville, Iowa; G. E. Frye, Fort Wayne, Ind.; W. C. Giller, Roodhouse, Ill.; F. W. Goodsall, Ottawa, Ill.; F. A. Goodbody, Chicago, Ill.; John P. Graff, New Ulm, Minn.; H. H. Glenn, Verona, Ill.; J. L. Halloran, Stapleton, N. Y.; J. H. Hanna, Burlington, Kas.; W. L. Hyatt, Erie, Kas.; N. W. Hillock, Columbus, Ohio; A. C. Howe, Des Moines, Iowa; W. F. Hoehner, Belleville, Ill.; J. K. Jameson, Paris, Ky.; E. C. Joss, Fairview, Kas.; G. A. Kay, Minden, Iowa; W. J. Kirk, Sharon, Pa.; G. W. Knorr, Louisville, Ky.; F. A. Laird, Springfield, Ill.; F. Lett, Jr., Paris Crossing, Ind.; W. H. Luther, Boonville, Ind.; R. C. Lew, Highland, Ill.; C. D. Maulfair, Magnolia, Ill.; R. Mazza, Petaluma, Cal.; A. F. Nelson, Jamestown, Ind.; R. E. Nesbitt, Maroa, Ill.; C. L. Passmore, Huntley, Ill.; P. J. Purcell, Bradford, Pa.; G. A. Rohde, Flint, Mich.; C. R. Richards, Victoria, B. C.; T. A. Schneekloth, Pepin, Wis.; F. K. Scott, Terra Haute, Ill.; C. O. Seaberg, Crystal Falls, Mich.; C. H. Spangler, Lockport, Ill.; M. A. Stewart, Richmond, Ind.; S. P. Talbot, Ames, Iowa; C. D. Tuttle, Canton, S D.; F. H. Thompson, Woolley, Wash.; G. S. Throp, Palestine, Ohio; C. O. Van Winkle, Salem, Iowa; C. A. Webber, Rochester, N. Y.; L. E. Warner, Aurora, Ill.

Of this number the following gentlemen graduated with honors: B. F. Barber, F. W. Brewer, W. L. Hyatt, E. C. Joss, W. J. Kirk, George W. Knorr, W. H. Luther, A. F. Nelson, C. R. Richards, C. O. Van Winkle, L. E. Warner, C. H. Spangler, C. A. Webber.

Dr. Edward C. Joss obtained the gold medal for the highest general average; Dr. C. R. Richards, the gold medal for the highest standing in equine theory and practice; Dr. George W. Knorr, the gold medal for the highest standing in anatomy; Dr. C. O. Van Winkle received the gold medal for the highest standing in cattle pathology. Dr. C. R. Richards also received the prize for the best examination in surgery; Dr. E. C. Joss, prizes for the best examinations in meat-inspection and helminthology; Dr. C. A. Webber, the prize for the highest standing in pathology and bacteriology; Dr. W. H. Luther, the prize in materia medica; Dr. C. H. Spangler, the prize in lameness; Dr. J. Frank, the prize for the highest average in physiology; Dr. T. A. Schneekloth, the prize for the highest standing in hygiene; Dr. C. J. Dawdy, the prize for the highest average in chemistry.

The degree of Doctor of Comparative Medicine (M.D.C.) was then conferred on the class by Prof. A. H. Baker, and the diplomas were distributed by Prof. E. L. Quitman.

Following the distribution of diplomas came the awarding of medals and prizes, after which the valedictory was delivered by Dr. J. H. Hanna, and was heartily applauded.

Prof. A. H. Baker delivered the doctorate, wishing the students Godspeed, and alluded to the fine prospects of the veterinary profession as evinced by the numberless requests from all parts of the country for veterinary surgeons received during the past session, as well as by the large number of students who registered at the Chicago Veterinary College; and giving them advice and encouragement, and also impressing the fact on the graduating class to always consider every member of the Faculty as their friend, and not to hesitate, whenever advice was necessary, to turn to their alma mater.

